# Occupational stress: evidence from industries affected by COVID-19 in Japan

**DOI:** 10.1186/s12889-022-13257-y

**Published:** 2022-05-18

**Authors:** Xiangdan Piao, Jun Xie, Shunsuke Managi

**Affiliations:** 1 Faculty of Humanities and Social Science, Iwate University, 3-18-34 Ueda, Morioka, Iwate, 020-8550 Japan; 2 Urban Institute & Department of Civil Engineering, Kyushu University, 744 Motooka Nishi-ku, Fukuoka, 819-0395 Japan

**Keywords:** COVID-19, Japan, Occupational stress, Industry

## Abstract

**Background:**

This study provides objective evidence on the impact of COVID-19 based on employee occupational stress reported from 13 different industries, and examines the determinants of employee psychological well-being. As the economic and social impacts of the COVID-19 pandemic continue, governments should consider industry-level differences when making support decisions concerning public resource allocation to corporations. However, little evidence exists regarding the differences in occupational stress across industries.

**Methods:**

Employee occupational stress data (*N* = 673,071) was derived from workers in Japan from 2018 to 2020. The sample comprises workers from 13 industries, including civil services, service industry (other), real estate, medical/welfare, wholesale/retail, academic research, and accommodation/restaurant business. A logit model is employed to investigate the differences in employees’ psychological well-being before and during the pandemic.

**Results:**

In 2020, 11 out of 12 industries had significantly worse occupational stress compared to employees engaged in civil services. Over 23% of employees from the wholesale/retail and accommodation/restaurant industries were observed as high-stress employees. Improved compensation policies supporting these industries are suggested. In contrast, reduced occupational stress was found among employees in the transportation/postal and information/communication industries. Among the 13 industries, aside from high job demands, tough inter-person relationships in the workplace became the most significant stressors during the pandemic.

**Conclusions:**

The results confirm that the pandemic has had a heterogeneous effect on employee occupational stress across industries, thus suggesting that the level of compensation given to different industries during the COVID-19 pandemic should be discussed and approved by the Japanese government. Additionally, support for the wholesale/retail and accommodation/restaurant industries during the pandemic should be improved.

**Supplementary Information:**

The online version contains supplementary material available at 10.1186/s12889-022-13257-y.

## Background

The rapid spread of COVID-19 has caused unprecedented global recession. As such, both healthcare crises and economic burdens engender occupational stress across industries. High occupational stress has been found to potentially change the career choice of employees across industries [1], which may result in large transformations in the job market and industry structure. Given both the short- and long-term economic and social impacts of the pandemic, industry-level differences need to be considered during the decision-making process when determining public resource allocation and effective plans to support the mental health of workers. However, there is little evidence indicating the differences in occupational stress across industries. This study aims to determine employee occupational stress levels prior to and during the COVID-19 pandemic by comparing the psychological well-being of employees from 12 industries and civil service, as the effect of the pandemic might differ across industries. Employee psychological well-being is measured using occupational stress and the share of high-stress employees in different industries. Moreover, the determinants of high stress employees are estimated for each industry.

Many studies have focused on occupational stress in the healthcare sector, where employees are working at the frontline and have been directly affected by the pandemic [2, 3]. Healthcare workers’ mental health has worsened during the pandemic, the impacts of which are believed to persist even after the pandemic. Factors such as the shortage of personal protective equipment, mortality and morbidity associated with COVID-19, fear of spreading the virus to family members, and the reality of losing colleagues to the disease are found to have significant effects on healthcare workers’ mental health [2]. Particularly, female health professionals have experienced high levels of stress during the pandemic, and the main stressors are related to safety concerns, staff and resource adequacy, workload and compensation, and job roles and security [3].

Moreover, global efforts exerted to prevent the spread of the disease, such as lockdowns, travel restrictions across countries and regions, school and factory closures, and social distancing, have caused an unprecedented global economic recession. Most industries have suffered during this pandemic, and the widespread panic has profoundly affected the mental health of employees working in the impacted industries. For instance, a study by Wong and colleagues [4] found that hotel employees’ pre-pandemic perceptions of occupational stressors differed from their perceptions since the onset of the pandemic [4]. With a surge in underemployment rates during the pandemic, traditional hotel work stressors did not worsen job satisfaction or organizational commitment [4], while unstable and more demanding hotel environment stressors and unethical labor practices following the onset of COVID-19 appeared to be the main triggers of lower job satisfaction and organizational commitment. In line with these results, studies also found that COVID-19-induced layoffs have increased disease-related stress among survivors, which in turn have decreased job performance. Notably, perceived organizational support against COVID-19 did not improve this situation [5].

Working restaurant employees have also been found to experience higher psychological distress compared with furloughed employees. Issues pertaining to increased substance use and the desire to seek future employment in alternate industries provides insights about social stability and potential job market changes [1]. Regarding COVID-19-induced stress, studies conducted in Japan have typically focused on the general population, and they have documented that relationships with people have had a more significant impact on stress [6].

Theoretical and empirical studies have effectively examined factors influencing employees’ occupational stress. For example, the job demand resource model suggests that greater job demand with low authority over job process control is associated with the likelihood of psychological illness among workers [7, 8]. Other influencing factors have been pointed out in prior research, including surround support systems, psychological impacts of tough job demands, unbalanced effort–reward ratio, job insecurity, work-life conflict, and harassment [9–23].

This study aims to contribute to the current literature along the following aspects. First, an attempt is made to show the changes to the psychological well-being of employees prior to and following the onset of COVID-19, specifically by comparing the well-being of employees in 12 different industries with that of civil servants in Japan. This information can be used as objective evidence when evaluating government compensation policy toward industries substantially affected by the pandemic. The results are based on a large individual sample of employees in Japan, and the high response rate (around 85%) to the stress survey strengthens the reliability of the study findings. Details on employee occupational stress were derived from 13 major industries between 2018 to 2020, enabling an assessment of the transition of employees’ psychological well-being and their vulnerability to mental health concerns.

Second, the differences between employee functioning in the 13 major industries and the determinants of high stress among employees were explored in the analysis. To the best of our knowledge, this is the first study to compare the magnitude of the pandemic’s impact on employee occupational stressors (e.g., job demand) across industries. The results are expected to provide insightful evidence for policymakers on workplace environment improvement.

## Materials and methods

### Participants

This study examined the effects of the COVID-19 pandemic on employee psychological well-being across various industries in Japan. Occupational stress data for working professionals, provided by Social Advance Inc. Japan, was collected between 2018 to 2020. The study design was approved by the appropriate legal and ethics review board of the Social Advance Inc. Japan. The data was provided with informed consent, according to legal and ethical guidelines. All the methods were proceeded in accordance with ethical guidelines and approved by the ethical committee of Social Advance Inc. Japan associated with Kyushu University, Japan.

The data included the detailed psychological well-being status of employees, their perceived evaluation of their workplace, and their demographic information. According to the guideline of the Ministry of Health, Labour and Welfare, Japan [24], the government requires corporations with over 50 employees to conduct an employee psychological well-being survey each year through a third-party company. The special advantage of these data is the high response rate, which on an average, is around 85% of respondents in each company. In total, 673,071 valid observations were collected from 2018 to 2020. In 2020, 320,348 valid occupational stress observations were made, whereas in 2019, the sample size was 219,768. In 2018, 132,955 observations were derived.

### Occupational stress and high stress employee

The employees were required to respond to the questions listed in the questionnaire provided by the Ministry of Health, Labour and Welfare [24]. Feeling or experiencing of active; full of energy; lively; angry; inwardly annoyed or aggravated; irritable; extremely tired; exhausted; weary or listless; tense; worried or insecure; restless; depressed; doing anything was a hassle; concentrate; gloomy; handle work; sad; dizzy; joint pains; headaches; stiff neck and / or shoulders; lower back pain; eyestrain; heart palpitations or shortness of breath; stomach and / or intestine problems; appetite; diarrhea and / or constipation; sleep [17]. The full detailed questionnaire is displayed in Additional file 1. Each question had the same answer choices: almost never = 1, sometimes = 2, often = 3, and almost always = 4. Occupational stress is a summation of the values chosen for the 29 question items (see the Additional file 1). The occupational stress score ranged between 29 to 116, with larger scores indicating poorer employee stress levels. The items “energetic,” “cheerful,” and “lively” were reverse scored. As per the Ministry’s guideline for the Brief Job Stress Questionnaire, an employee that satisfied either condition of the following two standards was categorized as a high-stress employee: (1) an employee occupational stress score greater than 77, or (2) occupational stress scores related to evaluation of workplace and surrounding support greater than 63 and 77, out of 116, respectively.

The industry classification dummies in this study included civil services, service industry (other), real estate, medical/welfare, wholesale/retail, academic research, professional/technical service, accommodation/restaurant business, construction, information/communication, education, manufacturing, and transportation/postal services.

### Covariates

Other explanatory variables included female (yes = 2, no = 1), employees’ age, and company size. The company size was a continuous variable that measured the number of employees in each company.

### Workplace environment

The determinant factors of high workplace stress among employees comprised nine aspects of workplace environment and three aspects of surrounding support. The first five aspects of workplace environment included tough workload quantity, tough workload quality, tough workplace body burden, tough workplace interpersonal relationships, and awful physical workplace environment. The evaluation of these five work environment aspects ranged between 1 to 4, with greater scores indicating poorer work environment situations. The other four aspects of workplace evaluations included good job control in the workplace, appropriate use of individual skills at work, appropriate match of skills to the work content, and a good balance between work and reward. These four aspects were evaluated on a range of 1–4, with greater scores showing better workplace situations and a good match between the employee and their work. Surrounding supports for the employee consisted of support from the boss, colleagues, and family and friends. The evaluation of support ranged from 1 to 4, with greater values indicating better levels of support.

Table 1 describes the employee occupational stress among different industries between 2018 and 2020. The industries are ranked in order of employee occupational stress level, with smaller numbers indicating worse employee psychological well-being. We found that employees working in the wholesale/retail and accommodation/restaurant service industries have worse psychological well-being, whereas those employed in transportation and real estate had the lowest stress levels. Table 2 displays the descriptive statistics and the Cronbach’s α scores. All the scores are greater than 0.7, indicating the reliability of the items. The correlation analysis results for the variables are summarized in the Additional file 1. Table A1 displays Pearson’s correlation coefficients of among occupational stress and dependent variables.

**Table 1:** Employee occupational stress among 13 industries from 2018 to 2020

Industry	Rank	Employee occupational stress		
		2018	2019	2020
Civil Service	8	54.7	54.87	56.37
Service Industry (other)	11	54.33	57.25	55.86
Real Estate	13	55.1	55.15	54.28
Medical/Welfare	4	60.31	59.46	59.35
Wholesale / retail	1	58.27	59.34	63.34
Academic research, professional / technical service industry	12	57.46	57	55.81
Accommodation business, restaurant service business	2	61.75	58.43	62.13
Construction industry	7	57.16	56.84	56.5
Information and communication industry	5	58.73	60	57.9
Education, learning support business	6	56.54	57.37	56.94
Life-related service industry, entertainment industry	9	55.57	57.23	56.35
Manufacturing industry	3	60.72	60.93	59.49
Transportation, postal	10	58.43	57.93	56.02
		132,955	219,768	320,348

Data source: Employee occupational stress data, Social Advance Inc. 2018 ~ 2020

**Table 2:** Descriptive statistics

Variable	Obs	Mean	Std. Dev.	Cronbach’s alpha
Occupational stress	673,071	55.29	14.81	0.94
High stress employee	667,965	0.11	0.32	–
Tough workload quantity	672,132	2.79	0.73	0.77
Tough workload quality	671,974	2.89	0.64	0.74
Tough workload body burden	672,714	2.36	1.03	–
Tough workplace inter-person relationship	671,622	1.96	0.61	0.71
Awful physical workplace environment	672,595	2.16	0.91	–
Good job control	672,157	2.56	0.64	0.73
Use skills	672,611	2.96	0.75	–
Appropriate matching to work content	672,695	2.85	0.76	–
Work reward balance	672,741	2.99	0.79	–
Support from boss	671,765	2.56	0.73	0.84
Support from colleague	671,721	2.73	0.70	0.84
Support from family friend	671,602	3.26	0.71	0.88
Female	673,071	1.42	0.49	–
Age	673,071	42.68	13.19	–
Company size	673,071	10,499	8957	–

Data source: Employee occupational stress data, Social Advance Inc. 2018 ~ 2020. Table A1 displays Pearson’s correlation coefficients of among occupational stress and dependent variables

### Study design

This study illustrated differences in the psychological well-being of employees from various industries prior to and after the onset of the pandemic. The dependent variables that expressed employee psychological well-being were occupational stress and high stress employee. Occupational stress was a continuous variable that ranged from 29 to 116, with greater values indicating poorer psychological well-being (Eq. 2). High stress employee was a binary variable identified by employees based on the guideline put forward by the Japanese government (Eq. 3). When the dependent variable is a binary variable, the logit model is considered appropriate; however, since occupational stress is a continuous variable, the ordinary least squares method was utilized [23, 25]. First, the overall heterogeneity of occupational stress across industries was investigated, as shown in Eqs. (1) and (2). The logit models used for regressing are shown in Eqs. (3), (4) and (5).

1 $${S}_{it}={\theta}_0+{Y}_{it}{\theta}_1+{X}_{it}\delta +{D}_t\gamma +{\varepsilon}_{it}$$

2 $${S}_i={\theta}_0+{Y}_i{\theta}_1+{X}_i\delta +{\varepsilon}_i$$

where *S*_*i*_ denoted employee *i* ’s occupational stress, ranging between 29 to 116, with higher values indicating poorer psychological well-being. *t* denoted the year between 2018 to 2020. *Y* comprised a set of dummy variables capturing industry fixed effect, which includes medical, wholesale/retail, accommodation/restaurant, service industry (other), real estate, professional service, construction, education, entertainment, manufacturing, transportation/postal, information/communication, and professional services. *X* comprised the explanatory variables that affected employees’ industry selection and psychological well-being, including the female gender dummy, age, and company size. *D*_*t*_ was a set of year dummy variables. The estimated parameters were *θ*_0_, *θ*_1_, *δ*, and *γ*, and the error term was *ε*.

Comparisons of high employee stress across various industries were investigated using the logit model (Eq. 3).

3 $${H}_{it}={a}_0+{Y}_{it}{a}_1+{X}_{it}{a}_2+{D}_t\gamma +{\varepsilon}_{it}$$

4 $${H}_i={\alpha}_0+{Y}_i{\alpha}_1+{X}_i\beta +{\varepsilon}_i$$

Where *H*_*i*_ denoted employee *i* ’s high stress status (yes =1, no =0), based on the standard established by the Japanese governmental guidelines. *t* denoted the year between 2018 to 2020. *Y* referred to a set of industry dummy variables denoting the industry to which the employee belonged. *X* denoted the explanatory variables. *D*_*t*_ was a set of year dummy variables. The estimated parameters were *a*_0_, *a*_1_, *a*_2_, *α*_0_, *α*_1_, and *β*, and the error term was *ε*.

The relationship between employees’ high-stress status and determinant factors were investigated using Eq. (4), based on the logit model.

5 $${H}_i={\gamma}_0+{Z}_i{\gamma}_1+{\varepsilon}_i$$

Again, *H*_*i*_ referred to employee ’s high-stress status, whereas *Z* referred to the determinant factors evaluating the quality of the workplace environment. Separate regression analyses were performed for each industry for all the factors, which included workload quantity, tough workload quality, tough workplace body burden, tough workplace interpersonal relationships, awful physical workplace environment, good job control in the workplace, appropriate use of individual skills at work, appropriate match of skills with work content, balance between work and reward, and support from the boss, colleagues, and family and friends.

## Results

**Fig. 1:**
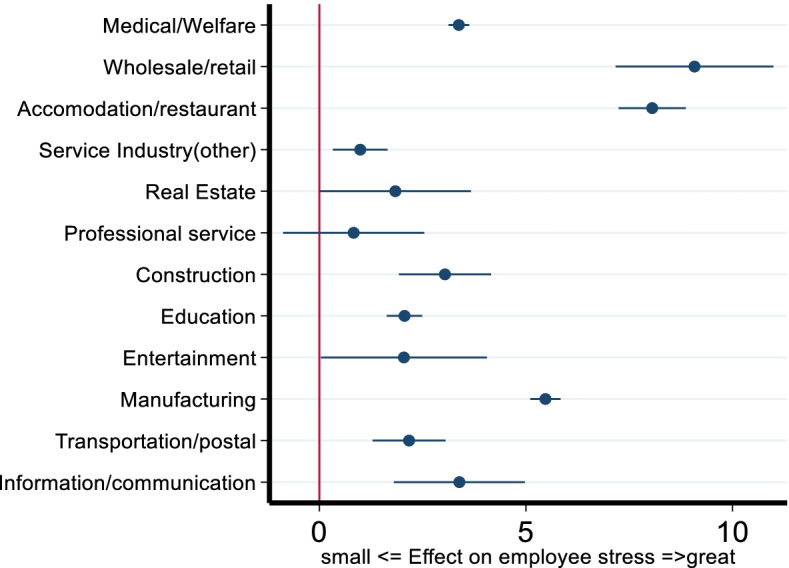
Comparison of occupational stress among employees belonging to civil services and other industries

Figure 1 illustrates the occupation comparisons between civil service employees and employees from other industries. The coefficients with 95% confidence intervals are estimated from Eqs. (1), (2), (3) and (4) and presented in Table 3**.** The coefficient for the medical/welfare industry was 3.376 and statistically significant, which suggests that employees in this industry have poorer psychological well-being compared to civil service employees. In fact, employees from 11 out of the 12 industries had significantly higher occupational stress scores than employees who served in the civil services industry in 2020. Particularly, employees who worked in the wholesale/retail and accommodation/restaurant industries showed worse psychological well-being, which requires further attention.

**Table 3:** Occupational stress and high stress among employees in each industry

	Occupational stress			High stress employee		
	Total	2020	2019	Total	2020	2019
Variables	(1)	(2)	(3)	(4)	(5)	(6)
**Industry dummies**	Coeff.(SE)	Coeff.(SE)	Coeff.(SE)	Coeff.(SE)	Coeff.(SE)	Coeff.(SE)
*Reference (civil service)*						
Medical	3.717***	3.376***	3.080***	0.050***	0.042***	0.042***
	(0.104)	(0.128)	(0.270)	(0.002)	(0.002)	(0.005)
Wholesale retail	3.475***	9.079***	3.721***	0.040***	0.111***	0.044***
	(0.171)	(0.973)	(0.257)	(0.003)	(0.013)	(0.005)
Accommodation/restaurant	7.914***	8.055***	4.998	–	0.096***	–
	(0.335)	(0.415)	(3.953)		(0.006)	
Service Industry(other)	0.332	0.991***	−2.158***	0.011**	0.013*	−0.011
	(0.269)	(0.337)	(0.487)	(0.006)	(0.007)	(0.011)
Real Estate	2.416***	1.840**	3.049*	0.028*	0.018	0.023
	(0.793)	(0.932)	(1.789)	(0.016)	(0.018)	(0.042)
Professional service	1.434**	0.832	1.407	−0.017	− 0.017	− 0.082
	(0.722)	(0.871)	(1.653)	(0.018)	(0.020)	(0.062)
Construction	3.355***	3.041***	3.105***	0.031***	0.021*	0.023*
	(0.320)	(0.570)	(0.535)	(0.006)	(0.011)	(0.012)
Education	1.929***	2.062***	1.610***	0.020***	0.020***	0.017***
	(0.139)	(0.219)	(0.193)	(0.003)	(0.004)	(0.004)
Entertainment	2.032***	2.047**	2.796*	0.008	0.001	−0.012
	(0.691)	(1.024)	(1.477)	(0.015)	(0.022)	(0.037)
Manufacturing	5.949***	5.471***	5.912***	0.070***	0.060***	0.070***
	(0.092)	(0.186)	(0.152)	(0.002)	(0.003)	(0.003)
Transportation/postal	3.557***	2.172***	4.050***	0.055***	0.032***	0.062***
	(0.277)	(0.450)	(0.384)	(0.005)	(0.009)	(0.007)
Information/communication	4.404***	3.390***	5.130***	0.051***	0.024	0.066***
	(0.433)	(0.809)	(0.735)	(0.008)	(0.015)	(0.012)
Female	3.890***	3.991***	4.071***	0.034***	0.033***	0.038***
	(0.037)	(0.053)	(0.065)	(0.001)	(0.001)	(0.001)
Age	−0.046***	−0.033***	− 0.060***	−0.000***	− 0.000***	−0.000***
	(0.001)	(0.002)	(0.002)	(0.000)	(0.000)	(0.000)
Company size	0.000***	−0.000***	0.000***	0.000***	−0.000***	0.000***
	(0.000)	(0.000)	(0.000)	(0.000)	(0.000)	(0.000)
Year dummies	Yes			Yes		
Observations	673,071	320,348	219,768	667,965	320,348	219,768
R-squared	0.031	0.029	0.031	0.0106	0.0106	0.0106

Data sources: Employee occupational stress data, Social Advance Inc. 2018 ~ 2020. The robust results are displayed in Table A2

*Coeff.* Coefficient, *SE* Robust Standard Error

*** *p* < 0.01, ** *p* < 0.05, * *p* < 0.1

**Fig. 2:**
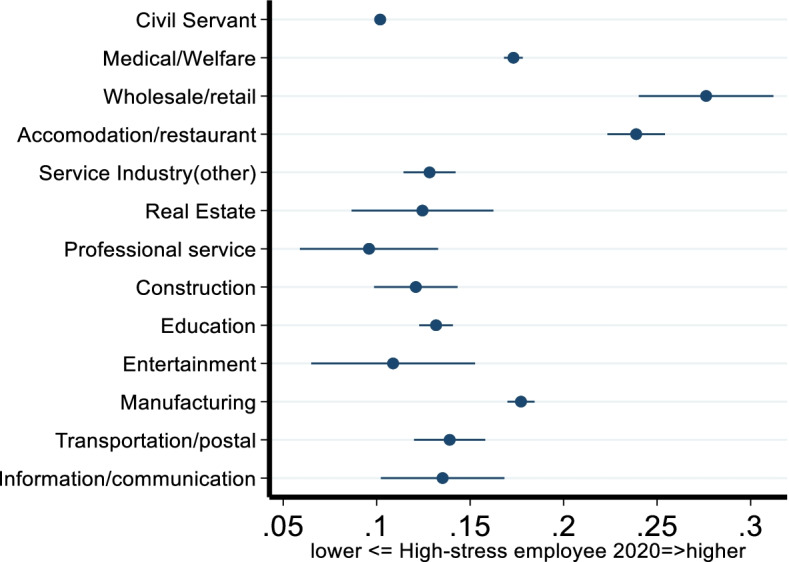
High stress among employees across industries in 2020

Figure 2 shows the comparison of high stress levels among employees (share of total employees) from civil services and other industries. High stress among employees are thought to be at increased risk of mental illness. It was found that in each industry, a moderate share of employees reported high stress levels. Precisely, across the industries, 10.2–27.6% of the employees demonstrated high stress, based on the occupational stress survey conducted in 2020. Employees who served in the wholesale/retail or accommodation/restaurant industries reported the worst psychological well-being when compared to other industries. It is notable that 27.6 and 23.9% of the employees were categorized as workers with high stress levels. The results suggest that among the industries, there is heterogeneous variance in psychological well-being, with wholesale/retail and accommodation/restaurant industries showing the worst levels of employee psychological well-being during the COVID-19 pandemic.

Table 3 presents the comparison of employees’ psychological well-being among industries in 2019 and 2020 to show differences in stress status prior to and following the onset of the pandemic. The results from Eqs. (1) and (2) are displayed in columns 1–3, with occupational stress as the dependent variable. The occupational stress scores ranged from 29 to 116, with greater values indicating worse stress levels. In columns 4–6, the results on high stress among employees are summarized by estimating Eqs. (2) and (4). The binary variable of the high-stress dummy equals 1 if the employee score falls under the psychological distress threshold, based on the Japanese governments’ guideline.

The results are summarized as follows. First, in the year 2020, during the pandemic, the employees working in the civil services sector appeared to have the best psychological well-being levels compared with employees from other industries. The coefficient value for the medical industry was 3.376 and statistically significant at the 1% level in 2020. The result suggests that employee stress for those working in the medical industry was, on average, 3.376 points worse than corresponding employees working in civil services. The industry coefficients were found to have positive values across the industries, namely, medical, wholesale/retail, accommodation/restaurant, service industry (other), real estate, professional service, construction, education, entertainment, manufacturing, transportation/postal, and information/communication, and the magnitudes were between 0.991 and 9.079. This indicated poorer psychological well-being among employees in these industries when compared to the corresponding group of civil servants. The results could be attributable to the pressure to make profits in the commercial sectors, leading to relatively higher stress compared to employees hired by the government for public services.

Regarding high stress levels among employees, it was found that employees belonging to the wholesale/retail and accommodation/restaurant industries required further attention from policy makers. The coefficient values for the wholesale/retail and accommodation/restaurant industries were 0.111 and 0.096, respectively, and both were statistically significant at the 1% level. The results indicate an 11.1% probability of higher stress levels, and 9.6% probability among employees in the wholesale/retail and accommodation/restaurant industries compared with those belonging to the civil services industry. Similarly, employees from the medical care, service, transportation/postal, and manufacturing industries were observed to have higher stress than those from the civil services industry, with a relatively moderate magnitude of high stress. The Japanese government declared the provision of financial aid for the wholesale/retail, accommodation/restaurant, and medical care industries on recognizing the vulnerability of these industries to financial problems during the COVID-19 pandemic. However, there is a lack of evidence on whether the aid policy introduced by the government has been sufficient. The results from this study are expected to provide insightful, objective evidence to aid in evaluating the impacts of the COVID-19 pandemic on different industries. The results suggest that financial aid from the government toward the accommodation/restaurant and wholesale/retail industries might be insufficient.

Comparing employee psychological well-being in 2019 and 2020, both improvements and aggregations across industries have been derived. For example, the coefficients of the wholesale/retail industry were 3.721 and 9.079, and both were statistically significant at the 1% level, which showed that employees belonging to the wholesale/retail industry appeared to have a substantial aggregation in their psychological well-being scores. Similar trends were observed in the accommodation/restaurant and service industries. In contrast, employees who served in the real estate, transportation/postal, and information/communication industries demonstrated better psychological well-being in 2020 than in 2019. This could be attributable to the introduction of tele-work by several corporations, thereby enabling employees to work comfortably from home. Notably, the magnitude of the coefficients for high stress levels among employees from the medical industry were the same at 0.042 and were statistically significant at the 1% level. This suggests that employees who serve in the medical industry have stable psychological well-being levels. This could be due to initiatives taken by the Japanese government to strengthen support for medical hospitals and improve the psychological well-being of doctors and nurses.

Table 4 presents the relationship between high stress levels among employees, the workplace environment, and surrounding support in 2020 during the COVID-19 pandemic. The dependent variable was the high stress level experienced by employees (yes =1, no =0). The results, derived from Eq. (5) using the logit model, for the 13 industries are displayed in Table 4.

**Table 4:** The relationship between high stress among employees and the workplace environment

	**(1)**	**(2)**	**(3)**	**(4)**	**(5)**	**(6)**	**(7)**
**Variables**	**Civil servant**	**Medical**	**Wholesale retail**	**Accommodation /restaurant**	**Service Industry(other)**	**Real Estate**	**Professional service**
*Workplace*							
Tough workload quantity	4.185***	5.578***	4.208***	4.694***	3.328***	3.742***	5.892***
	(0.026)	(0.360)	(0.890)	(0.131)	(0.166)	(0.891)	(0.429)
Tough workload quality	2.960***	1.228***	4.754***	2.887***	3.402***	2.419**	1.893***
	(0.030)	(0.390)	(1.015)	(0.150)	(0.182)	(0.948)	(0.433)
Tough workload body burden	0.362***	0.065	−1.537**	0.652***	0.921***	−0.361	1.535***
	(0.015)	(0.209)	(0.648)	(0.079)	(0.104)	(0.693)	(0.263)
Tough workplace inter-person relationship	3.896***	4.266***	3.495***	3.932***	4.088***	3.554***	5.176***
	(0.029)	(0.376)	(1.015)	(0.134)	(0.181)	(1.011)	(0.402)
Awful physical workplace environment	1.447***	1.882***	0.723	1.466***	1.429***	1.079	1.371***
	(0.017)	(0.226)	(0.673)	(0.085)	(0.102)	(0.660)	(0.240)
Good job control	− 2.311***	− 1.422***	−2.474**	− 2.705***	−2.102***	− 1.141	− 1.401***
	(0.026)	(0.335)	(0.976)	(0.123)	(0.166)	(0.944)	(0.371)
Use of possessed skills	−0.456***	0.387	−0.426	−0.488***	−0.115	−2.441***	− 0.137
	(0.022)	(0.262)	(0.693)	(0.100)	(0.133)	(0.736)	(0.297)
Appropriate matching to work content	−2.102***	−1.938***	− 1.493	− 2.872***	− 2.170***	−2.504***	−1.526***
	(0.026)	(0.356)	(0.907)	(0.126)	(0.162)	(0.925)	(0.376)
Work reward balance	−2.586***	− 2.415***	−3.522***	−2.107***	− 2.831***	−2.024**	−3.510***
	(0.026)	(0.351)	(0.921)	(0.126)	(0.161)	(0.953)	(0.349)
*Surrounding support*							
Support from boss	−1.278***	−1.514***	−2.891***	− 1.438***	− 1.431***	− 3.010***	−1.429***
	(0.028)	(0.357)	(0.999)	(0.122)	(0.167)	(1.035)	(0.386)
Support from colleague	−1.282***	− 1.376***	0.431	−0.844***	−0.879***	1.614	− 0.120
	(0.030)	(0.375)	(1.047)	(0.123)	(0.176)	(1.071)	(0.385)
Support from family friend	−2.051***	−1.767***	−0.990	−2.184***	− 1.754***	−3.488***	− 2.534***
	(0.022)	(0.286)	(0.769)	(0.105)	(0.142)	(0.702)	(0.293)
Constant	57.739***	53.467***	57.983***	58.159***	56.456***	68.629***	49.716***
	(0.157)	(1.972)	(4.963)	(0.793)	(1.007)	(5.319)	(2.275)
Observations	591,356	3514	498	28,591	15,390	473	3275
R-squared	0.446	0.436	0.460	0.435	0.422	0.458	0.447
	**(8)**	**(9)**	**(10)**	**(11)**	**(12)**	**(13)**	
**Variables**	**Construction**	**Education**	**Entertainment**	**Manufacturing**	**Transportation/postal**	**Information/communication**	
*Workplace*							
Tough workload quantity	3.950***	3.182***	4.319***	3.815***	4.037***	5.636***	
	(0.368)	(0.497)	(0.195)	(0.812)	(0.111)	(0.350)	
Tough workload quality	3.374***	4.443***	2.844***	2.508***	3.292***	1.735***	
	(0.408)	(0.589)	(0.222)	(0.829)	(0.122)	(0.368)	
Tough workload body burden	−0.457**	−0.013	0.311***	−0.193	−0.053	0.291	
	(0.215)	(0.361)	(0.117)	(0.452)	(0.064)	(0.201)	
Tough workplace inter-person relationship	5.065***	3.530***	3.962***	2.986***	4.988***	5.321***	
	(0.392)	(0.556)	(0.196)	(0.889)	(0.116)	(0.355)	
Awful physical workplace environment	1.453***	2.143***	1.451***	1.626***	1.136***	0.683***	
	(0.226)	(0.334)	(0.128)	(0.511)	(0.068)	(0.221)	
Good job control	−2.032***	−1.896***	−2.644***	−3.059***	−2.041***	−0.890***	
	(0.342)	(0.508)	(0.185)	(0.812)	(0.106)	(0.310)	
Use of possessed skills	−0.486*	−0.553	− 0.366***	0.153	− 0.592***	0.129	
	(0.268)	(0.383)	(0.134)	(0.587)	(0.083)	(0.257)	
Appropriate matching to work content	−1.740***	− 1.275**	−1.981***	− 1.886**	−1.930***	−2.965***	
	(0.369)	(0.530)	(0.196)	(0.765)	(0.101)	(0.327)	
Work reward balance	−3.333***	−2.990***	− 2.488***	−5.104***	− 2.763***	−2.712***	
	(0.348)	(0.510)	(0.196)	(0.709)	(0.102)	(0.310)	
*Surrounding support*							
Support from boss	−1.034***	−1.644***	−0.551***	0.345	−1.120***	−1.344***	
	(0.362)	(0.489)	(0.174)	(0.731)	(0.110)	(0.366)	
Support from colleague	−1.764***	−0.288	−1.453***	− 1.329	− 0.657***	− 0.595	
	(0.367)	(0.501)	(0.179)	(0.827)	(0.114)	(0.368)	
Support from family friend	−1.082***	−2.183***	−2.069***	−0.753	− 2.028***	− 1.825***	
	(0.285)	(0.394)	(0.150)	(0.651)	(0.087)	(0.273)	
Constant	55.214***	56.489***	57.978***	63.141***	55.495***	51.124***	
	(2.173)	(2.936)	(1.131)	(4.751)	(0.642)	(1.951)	
Observations	3493	1727	12,820	650	39,228	3621	
R-squared	0.453	0.443	0.431	0.386	0.416	0.438	

Coefficients and robust standard errors are displayed in Table. Robust standard errors in parentheses

*** *p* < 0.01, ** *p* < 0.05, * *p* < 0.1

The results are summarized as follows. First, among the industries, the factors of workplace environment and surrounding support significantly influenced employees’ psychological distress. The results are consistent with previous studies [9–23]. On the one hand, the tough workload quantity, quality, and workplace interpersonal relationships worsened employee well-being across all 13 industries. Similarly, awful physical workplace environments were found to have a negative impact on psychological well-being in 11 out of 13 industries. The factor of body burden caused by tough workload had a mixed effect on the high stress levels of employees. In the wholesale/retail and construction industries, body burden was found to have an improved effect on the workers’ high-stress status, whereas among civil servants, for example, body burden had a negative influence. This result may be because the work contents are quite different between industries.

On the other hand, good job control, appropriate usage of individual skills at work, appropriate match of skills with work content, and balance between work and reward tended to reduce the likelihood of high stress among workers. Improvement of employee control over the workload and appropriate matching of skills to the work content had significant effects in reducing high stress in 12 out of 13 industries. Regarding balance between work and reward, it was found to positively affect employee well-being across industries.

Surrounding support from the boss, colleagues, and family and friends tended to improve employee psychological well-being. For example, the coefficient of support from the boss was − 1.278 and statistically significant at the 1% level in the civil services industry. These results suggest that employees who received support from their bosses had decreased probability of experiencing high stress. The positive effect of surrounding support from the boss, colleagues, and family and friends on reducing employee stress was observed in 12 out of 13 industries. Hence, improvements to these forms of surrounding support systems could help relieve the stress levels of employees experiencing higher stress.

Second, a heterogeneous effect was found between the industries with regards to the workplace environment, surrounding support, and employee distress. The two factors that influenced employee distress are summarized. For civil servants and employees from the medical care, real estate, professional, service, construction, entertainment, transportation/postal, and accommodation/restaurant industries, the largest two factors that affected employee distress were found to be tough workload quantity and tough workplace interpersonal relationships. On the contrary, tough workload quality and quantity had the greatest effects on employee psychological well-being. In the service and education industries, tough workload quality and tough workplace interpersonal relationships had the greatest impacts on high stress levels among employees. Finally, for the manufacturing industry, improvements in balance between work and reward and tough workload quantity had the most influence on employee psychological well-being. In total, tough workplace interpersonal relationships was found to be the worst factor in terms of the impact on psychological well-being in 12 out of 13 industries, whereas tough workload quantity appeared to be the worst in 11 out of 13 industries. Balance in workload quantity and improvements in workplace interpersonal relationships could help reduce high stress among employees.

## Discussion and conclusion

How does employee occupational stress in Japan differ across industries before and during the pandemic? In this study, a heterogeneous analysis of employee psychological well-being is summarized. Changes prior to and with the onset of the pandemic were explored to provide insightful evidence on industry-variated effects of policies and the pandemic on employee well-being. Large-scale cross-sectional employee occupational data collected between the years 2018 to 2020 were used.

The results indicate that, among the industries, employees in civil services appeared to have the best psychological well-being levels; further, 11 out of 12 industries had significantly worse occupational stress levels, with magnitudes ranging from 0.991–9.079. Regarding high stress levels, which consequently increase the risk of mental illness, stress levels among employees in the wholesale/retail and accommodation/restaurant industries were found to be 27.6 and 23.9%, respectively. In contrast, a score of 10.2% was found among civil services employees. Poorer psychological well-being was observed among employees from the wholesale/retail and accommodation/restaurant industries during the COVID-19 pandemic.

On the contrary, improvements to employee psychological well-being were found across the real estate, transportation/postal, and information/communication industries during the COVID-19 pandemic. The introduction of tele-work by corporations and a comfortable working environment could be determinants of these results. Regarding the medical care industry, employee psychological well-being appeared stable, which may be attributable to initiatives by the Japanese government to strengthen support for medical hospitals and improve the psychological well-being of doctors and nurses.

Further, for the relationship between employee psychological well-being and workplace environment, it was found that workload quantity, quality, and tough workplace interpersonal relationships had the greatest impacts on employee stress and increased the likelihood of high stress among employees across all industries. Similarly, the factors of employee job control, appropriate usage of individual skills, and balance between work and reward appeared to reduce the probability of employees experiencing high stress. Multiple surrounding supports from bosses, colleagues, and family are expected to improve psychological well-being.

The policy implications with reference to the results of this study are as follows. It is suggested that the compensation to various industries during the COVID-19 pandemic be discussed and approved by the Japanese government. For example, pertaining to additional payment to the medical care system for the treatment of COVID-19 patients, as well as for accommodation/restaurant companies, compensation could be paid to the respective corporations during the pandemic emergency period. According to the results derived from this study, based on the variable of employee psychological well-being, a comprehensive policy developed to support the wholesale/retail and accommodation/restaurant industries during the pandemic should be improved. This is because the results show a tremendous increase in occupational stress, which persisted even after the commencement of financial aid. This suggests that economic burden is not the only issue faced by employees. Finally, this study provides some useful guidance for managers to develop plans for organizational support to alleviate occupational stress.

The limitations of this study are acknowledged as follows. First, the employee occupational stress data was collected in Japan. However, most of the companies surveyed are located in southern Japan rather than randomly collected nationwide. Therefore, to illustrate nationwide results, further studies based on comprehensive data randomly collected around the country should be conducted. Second, this study focused on comparing occupational stress among employees of various industries prior to and during the COVID-19 pandemic based on cross-sectional data. To more accurately identify those most in need of aid during a pandemic, future studies should rely on more comprehensive panel data collected prior to and during the pandemic. As for the research design, the limitation of the dataset did not allow us to conduct a panel data analysis as suggested. The dataset is repeated cross-sectional data, in which using the whole dataset can hardly capture the changes at the individual level prior to and following the onset of the pandemic. That is also the reason why we focus on the changes at the sectoral level and compare the heterogeneity among sectors, which is another critical issue regarding policymaking. As suggested, using the whole dataset will be ideal, and we hope that future research can obtain more detailed panel data to deal with the current limitation.

## Supplementary Information

**Additional file 1: Table A1.** Pearson's correlation coefficients of among occupational stress and dependent variables. **Table A2.** Occupational stress and high stress among employees in each industry—Robustness check.

## Data Availability

The data is publicly unavailable; however, the data is only accessible when Social Advance Inc. provides data access permission.
