# Supplementary material for: Occupational stress: evidence from industries affected by COVID-19 in Japan
Source: BMC Public Health. 2022 May 18;22:1005. doi: 10.1186/s12889-022-13257-y (PMC9116494; doi:10.1186/s12889-022-13257-y)
Supplement: Supplementary file 1 — Additional file 1: Table A1. Pearson's correlation coefficients of among occupational stress and dependent variables. Table A2. Occupational stress and high stress among employees in each industry—Robustness check. [file 12889_2022_13257_MOESM1_ESM.docx]

**Appendix**

**A. Occupational stress**

The employees were required to respond to the questions listed in the questionnaire provided by the Ministry of Health, Labour and Welfare [22]. Feeling or experiencing of active; full of energy; lively; angry; inwardly annoyed or aggravated; irritable; extremely tired; exhausted; weary or listless; tense; worried or insecure; restless; depressed; doing anything was a hassle; concentrate; gloomy; handle work; sad; dizzy; joint pains; headaches; stiff neck and / or shoulders; lower back pain; eyestrain; heart palpitations or shortness of breath; stomach and / or intestine problems; appetite; diarrhea and / or constipation; sleep [17]. Each question had the same answer choices: almost never = 1, sometimes = 2, often = 3, and almost always = 4. Occupational stress is a summation of the values chosen for the 29 question items. The occupational stress score ranged between 29 to 116, with larger scores indicating poorer employee stress levels. The items “energetic,” “cheerful,” and “lively” were reverse scored. As per the Ministry’s guideline for the Brief Job Stress Questionnaire, an employee that satisfied either condition of the following two standards was categorized as a high-stress employee: (1) an employee occupational stress score greater than 77, or (2) occupational stress scores related to evaluation of workplace and surrounding support greater than 63 and 77, out of 116, respectively.

The full detailed questionnaire for occupational stress is as follows. I have been very active; 2) I have been full of energy; 3) I have been lively; 4) I have felt angry; 5) I have been inwardly annoyed or aggravated; 6) I have felt irritable; 7) I have felt extremely tired; 8) I have felt exhausted; 9) I have felt weary or listless; 10) I have felt tense; 11) I have felt worried or insecure; 12) I have felt restless; 13) I have been depressed; 14) I have thought that doing anything was a hassle; 15) I have been unable to concentrate; 16) I have felt gloomy; 17) I have been unable to handle work; 18) I have felt sad; 19) I have felt dizzy; 20) I have experienced joint pains; 21) I have experienced headaches; 22) I have had a stiff neck and / or shoulders; 23) I have had lower back pain; 24) I have had eyestrain; 25) I have experienced heart palpitations or shortness of breath; 26) I have experienced stomach and / or intestine problems; 27) I have lost my appetite; 28) I have experienced diarrhea and / or constipation; 29) I have not been able to sleep well.

**Table A1: Pearson's correlation coefficients of among occupational stress and dependent variables**

|  | Occupational stress | High stress employee | Tough workload quantity | Tough workload quality | Tough workload body burden | Tough workplace inter-person relationship | Awful physical workplace environment | Well job control | Use skills |
| --- | --- | --- | --- | --- | --- | --- | --- | --- | --- |
| Occupational stress | 1 |  |  |  |  |  |  |  |  |
| High stress employee | 0.6666 | 1 |  |  |  |  |  |  |  |
| Tough workload quantity | 0.3946 | 0.2613 | 1 |  |  |  |  |  |  |
| Tough workload quality | 0.337 | 0.2433 | 0.6406 | 1 |  |  |  |  |  |
| Tough workload body burden | 0.1242 | 0.1129 | 0.2775 | 0.3202 | 1 |  |  |  |  |
| Tough workplace inter-person relationship | 0.4744 | 0.368 | 0.2315 | 0.1906 | 0.1087 | 1 |  |  |  |
| Awful physical workplace environment | 0.3176 | 0.225 | 0.1754 | 0.1388 | 0.0937 | 0.3265 | 1 |  |  |
| Good job control | -0.4053 | -0.3237 | -0.2409 | -0.2462 | -0.1473 | -0.3591 | -0.2209 | 1 |  |
| Use skills | -0.2054 | -0.1634 | 0.0531 | 0.112 | 0.0847 | -0.238 | -0.1549 | 0.2115 | 1 |
| Appropriate matching to work content | -0.4019 | -0.2941 | -0.1302 | -0.0743 | 0.0992 | -0.3184 | -0.1938 | 0.3594 | 0.3737 |
| Work reward balance | -0.3761 | -0.2903 | 0.0308 | 0.0944 | 0.1736 | -0.3564 | -0.2307 | 0.3139 | 0.3952 |
| Support from boss | -0.3715 | -0.2806 | -0.0589 | -0.0492 | -0.0348 | -0.4588 | -0.2404 | 0.3462 | 0.2149 |
| Support from colleague | -0.3579 | -0.2716 | -0.0315 | -0.0273 | 0.0424 | -0.429 | -0.207 | 0.2889 | 0.2275 |
| Support from family friend | -0.2834 | -0.2213 | -0.0661 | -0.0312 | 0.0334 | -0.2423 | -0.1402 | 0.154 | 0.1424 |
| Female dummy | 0.1294 | 0.0519 | 0.0695 | 0.0664 | 0.0851 | -0.0092 | 0.0361 | -0.0719 | 0.035 |
| Age | -0.0526 | -0.0201 | -0.1312 | -0.0763 | -0.1049 | 0.0142 | 0.0308 | -0.0113 | -0.0132 |
| Company size | -0.0251 | -0.0203 | 0.0374 | 0.0289 | 0.0229 | -0.0505 | -0.0126 | 0.0213 | 0.0086 |
|  |  |  |  |  |  |  |  |  |  |
|  | Appropriate matching to work content | Work reward balance | Support from boss | Support from colleague | Support from family friend | Female dummy | Age | Company size |  |
| Appropriate matching to work content | 1 |  |  |  |  |  |  |  |  |
| Work reward balance | 0.6581 | 1 |  |  |  |  |  |  |  |
| Support from boss | 0.2774 | 0.3604 | 1 |  |  |  |  |  |  |
| Support from colleague | 0.2937 | 0.3721 | 0.6649 | 1 |  |  |  |  |  |
| Support from family friend | 0.191 | 0.2254 | 0.3257 | 0.4247 | 1 |  |  |  |  |
| Female dummy | 0.0249 | 0.0519 | -0.0798 | 0.0139 | 0.078 | 1 |  |  |  |
| Age | 0.0217 | -0.0643 | -0.1304 | -0.1545 | -0.0831 | -0.0522 | 1 |  |  |
| Company size | 0.018 | 0.0493 | 0.0493 | 0.0246 | 0.0131 | -0.0381 | 0.0287 | 1 |  |

**Data source: Employee occupational stress data, Social Advance Inc. 2018~2020.**

**Table A2: Occupational stress and high stress among employees in each industry—Robustness check**

|  | Total | 2018 | 2018 |
| --- | --- | --- | --- |
| VARIABLES | Occupational stress | High-stress | Occupational stress |
|  | Coeff.(SE) | Coeff.(SE) | Coeff.(SE) |
| **Industry dummies** |  |  |  |
| *Reference (civil servant)* |  |  |  |
| Medical | 3.653*** | 0.077*** | 5.101*** |
|  | (0.175) | (0.004) | (0.242) |
| Wholesale retail | 3.227*** | 0.036*** | 3.086*** |
|  | (0.170) | (0.005) | (0.234) |
| Accommodation/restaurant | 7.110*** |  | 7.625*** |
|  | (0.493) |  | (0.569) |
| Service Industry(other) | -1.375*** | 0.043** | 1.217 |
|  | (0.425) | (0.019) | (1.022) |
| Real Estate | 2.408 | 0.064 | 1.887 |
|  | (1.673) | (0.063) | (2.505) |
| Professional service | 1.619 | 0.010 | 2.867 |
|  | (1.451) | (0.048) | (1.936) |
| Construction | 3.255*** | 0.059*** | 4.181*** |
|  | (0.390) | (0.011) | (0.553) |
| Education | 1.657*** | 0.032*** | 2.458*** |
|  | (0.178) | (0.010) | (0.491) |
| Entertainment | 1.572* | 0.034 | 1.879 |
|  | (0.905) | (0.024) | (1.187) |
| Manufacturing | 5.966*** | 0.086*** | 6.550*** |
|  | (0.101) | (0.003) | (0.148) |
| Transportation/postal | 3.977*** | 0.096*** | 5.284*** |
|  | (0.333) | (0.015) | (0.854) |
| Information/communication | 4.587*** | 0.068*** | 4.824*** |
|  | (0.477) | (0.013) | (0.709) |
| Female dummy | 3.893*** | 0.025*** | 3.071*** |
|  | (0.037) | (0.002) | (0.081) |
| Age | -0.046*** | -0.000*** | -0.054*** |
|  | (0.001) | (0.000) | (0.003) |
| Company size | 0.000*** | 0.000*** | 0.000*** |
|  | (0.000) | (0.000) | (0.000) |
| Service Industry(other)×2020 | 2.734*** |  | 51.880*** |
|  | (0.536) |  | (0.178) |
| Real Estate×2020 | 0.025 |  |  |
|  | (1.902) |  |  |
| Medical×2020 | 0.104 |  |  |
|  | (0.212) |  |  |
| Wholesale retail×2020 | 6.318*** |  |  |
|  | (0.875) |  |  |
| Professional service×2020 | -0.241 |  |  |
|  | (1.697) |  |  |
| Accommodation/restaurant×2020 | 1.264** |  |  |
|  | (0.616) |  |  |
| Construction×2020 | 0.283 |  |  |
|  | (0.661) |  |  |
| Education×2020 | 0.679** |  |  |
|  | (0.281) |  |  |
| Entertainment×2020 | 1.075 |  |  |
|  | (1.384) |  |  |
| Manufacturing×2020 | -0.103 |  |  |
|  | (0.201) |  |  |
| Transportation/postal×2020 | -1.220** |  |  |
|  | (0.563) |  |  |
| Information/communication×2020 | -0.713 |  |  |
|  | (0.923) |  |  |
| 2019.year | 0.197*** |  |  |
|  | (0.051) |  |  |
| 2020.year | -0.727*** |  |  |
|  | (0.050) |  |  |
| Constant | 51.239*** |  |  |
|  | (0.092) |  |  |
|  |  |  |  |
| Observations | 673,071 | 132,566 | 132,955 |
| R-squared | 0.031 |  | 0.036 |

**Data source: Employee occupational stress data, Social Advance Inc. 2018~2020. Standard errors in parentheses. *** p<0.01, ** p<0.05, * p<0.1.**
